# Class III-specific HDAC inhibitor Tenovin-6 induces apoptosis, suppresses migration and eliminates cancer stem cells in uveal melanoma

**DOI:** 10.1038/srep22622

**Published:** 2016-03-04

**Authors:** Wei Dai, Jingfeng Zhou, Bei Jin, Jingxuan Pan

**Affiliations:** 1 State Key Laboratory of Ophthalmology, Zhongshan Ophthalmic Center, Sun Yat-sen University; Jinan University Institute of Tumor Pharmacology, Guangzhou, China; 2 Collaborative Innovation Center for Cancer Medicine, State Key Laboratory of Oncology in South China, Sun Yat-Sen University Cancer Center, Guangzhou, China

## Abstract

Uveal melanoma (UM) is the most common intraocular malignancy in adults. Despite improvements in surgical, radiation and chemotherapy treatments, the overall survival of UM and prognosis remain poor. In the present study, we hypothesized that Sirtuin 1 and 2 (SIRT1/2), class III histone deacetylases (HDACs), were critical in controlling the destiny of bulk tumor cells and cancer stem cells (CSCs) of UM. We testified this hypothesis in four lines of UM cells (92.1, Mel 270, Omm 1 and Omm 2.3). Our results showed that inhibition of SIRT1/2 by Tenovin-6 induced apoptosis in UM cells by activating the expression of tumor suppressor genes such as p53 and elevating reactive oxygen species (ROS). Tenovin-6 inhibited the growth of UM cells. Tenovin-6 and vinblastine was synergistic in inducing apoptosis of UM cell line 92.1 and Mel 270. Furthermore, Tenovin-6 eliminated cancer stem cells in 92.1 and Mel 270 cells. In conclusion, our findings suggest that Tenovin-6 may be a promising agent to kill UM bulk tumor cells and CSCs.

Uveal melanoma (UM) is the most common primary intraocular malignancy in adults with an incidence of 5.1 per million, accounting for about 3% of all melanomas^1^. The etiology and biological pathways are poorly understood. The tumor biology of UM is quite distinct from that of cutaneous melanoma^2^. The cutaneous melanoma associated risk factors such as ultraviolet radiation does not correlate with UM^3^. Traditional treatment of primary lesions is enucleation of the affected eye. Other therapeutic options that may preserve vision include radiotherapy, phototherapy and systemic chemotherapy. Despite multiple treatment modalities, survival has not improved by much in the last five decades^2^. About 50% of patients with UM have metastasis particularly to the liver^2^. Once metastasis occurs, the prognosis of UM patients becomes poor with a median survival of about 10–18 months^4^.

The poor efficacy of treatment for primary lesions and metastasis is partially due to the lack of valid therapeutic targets. Instead of common occurence of BRAF or NRAS mutations in cutaneous melanoma, few cases of UM harbor BRAF and NRAS mutations^5^. Mutations in SF3B1 encoding subunit 1 of the splicing factor 3b protein which is a component of the U2 small nuclear ribonucleoprotein complex (snRNP) were observed to be associated with good prognosis and were rarely coexist with BAP1 mutations^6^. Additionally, C-Met kinase may be a promising therapeutic target for UM^7^,^8^.

Recent mutational profiling studies of UM have identified mutually exclusive activating mutations (e.g., Q209 and R183) in the two G protein coupled receptor (GPCR) alpha subunits, GNAQ and GNA11, and these are driver mutations in more than 80% of profiled UM tumors^9^. However, there are no effective inhibitors available for GPCR signaling.

The downstream targets of GPCR pathway activation include protein kinase C (PKC) and mitogen-activated protein kinase (MAPK or MEK)^10^,^11^. Recently, it has been demonstrated that the activating mutations in GPCR can inhibit large tumor suppressor kinases LATS1/2 and promote actin polymerization, both of which can eventually trigger accumulation of dephosphorylated (active) YAP in the nucleus and YAP-dependent transcription^12^. However, the benefit of inhibitors of the PKC-MEK pathway and the YAP pathway in patients with UM remains to be determined. Therefore, there is an urgent need to evaluate novel targets and develop corresponding therapeutic agents for UM.

Chromatin remodeling due to the alteration of histone acetylation tightly controls cell fate by regulating gene expression^13^. The status of histone acetylation is dependent on the balance of histone acetyltransferase (HAT) (e.g., PCAF, CBP, p300, Tip60 and MOF) activity and histone deacetylase (HDAC) (e.g., mSin3a, NCoR/SMRT and Mi-2/NuRD) activity^14^. Pan-HDAC inhibitors (HDACis) (e.g., Valproic acid, trichostatin A, LBH589)^15^, and Class II-specific HDACis (e.g., MC1586, MC1575)^16^ have showed potent antitumor activity in UM.

Sirtuin 1 and 2 (SIRT1/2), class III HDACs, are involved in a wide variety of cellular processes, including cell cycle, DNA repair and cell survival under stress conditions^17^. Overexpression of SIRT1/2 has been shown to predict poor prognosis in a wide variety of solid tumors such as pancreatic cancer^18^, non-small cell lung cancer^19^, and malignant hematological diseases such as chronic myeloid leukemia^20^ and acute lymphoblastic leukemia^21^. SIRT1/2 can promote resistance to conventional chemotherapeutic agents^19^,^22^. However, little is known about the role of SIRT1/2 in UM.

In the present study, we hypothesized that SIRT1/2 was critical in controlling the destiny of bulk tumor cells and cancer stem cells (CSCs) of UM, and that inhibiting SIRT1/2 by Tenovin-6 might result in apoptosis in UM cells by releasing expression of tumor suppressor genes such as p53 and elevating reactive oxygen species (ROS). We examined four lines of UM cells (92.1, Mel 270, Omm 1, and Omm 2.3). Our findings imply that Tenovin-6 is a promising agent to kill UM bulk tumor cells and CSCs.

## Results

### Tenovin-6 inhibits deacetylation activity of SIRT1/2 in UM cells

**Figure 1:**
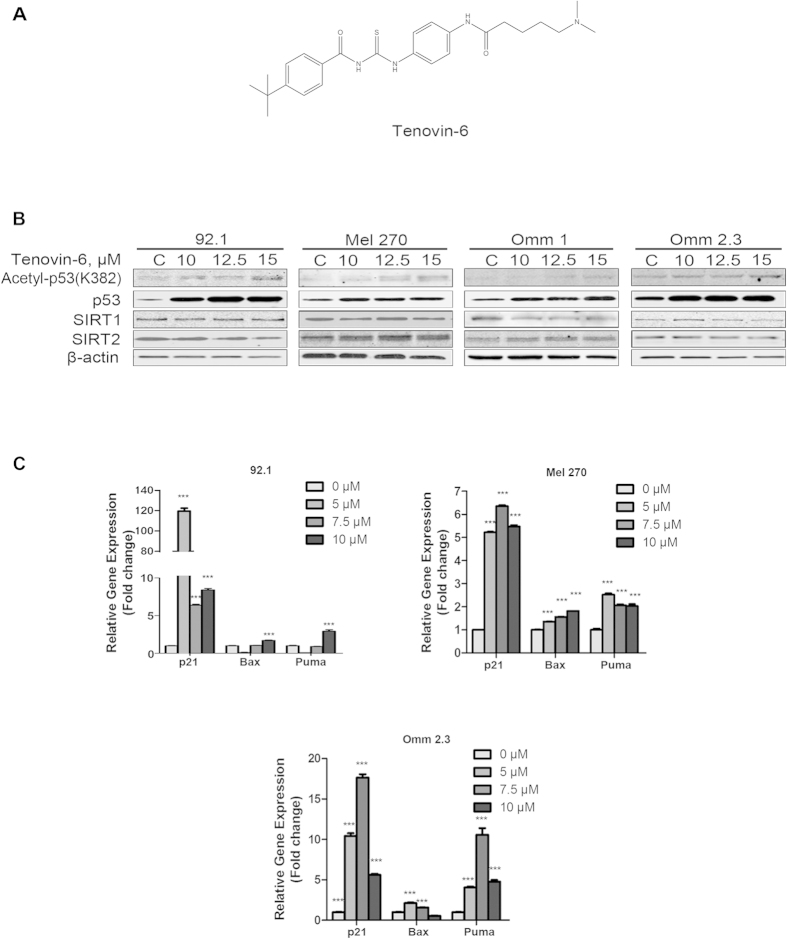
Tenovin-6 activates p53 in uveal melanoma cells. (**A**) Chemical structure of SIRT1/2 inhibitor Tenovin-6. (**B**) Uveal melanoma cells were treated with increasing concentrations of Tenovin-6 for 48 h. Western blotting analysis of acetylated p53, total p53 protein, SIRT1 and SIRT2 was performed after probing with the specific antibodies as labeled. (**C**) Uveal melanoma cells were exposed to escalating concentrations of Tenovin-6 for 48 h, and mRNA levels of p53 and its target genes (p21, Bax, and Puma) were examined by real-time PCR. GAPDH was used as an internal standard.

Our previous studies and other’s have shown that Tenovin-6 inhibits the deacetylation activity of SIRT1 and SIRT2 in diverse types of cancer cells^21^,^23^. To evaluate the effect of Tenovin-6 on SIRT1/2 in UM cells, four lines of UM cells (92.1, Mel 270, Omm 1 and Omm 2.3) were exposed to increasing concentrations of Tenovin-6 for 48 h, respectively. The whole cell lysates were then analyzed by Western blotting. The results showed that the levels of acetylated p53 and total p53 protein were increased in a concentration-dependent manner in 92.1, Mel 270, Omm 1 and Omm 2.3 cells (Fig. 1B).

We next examined the response of increased p53 by assessing the transcription of genes of p21, Bax and Puma , important downstream target genes of p53. Quantitative reverse transcriptase-polymerase chain reaction (qRT-PCR) analysis showed that the levels of mRNA of downstream target genes of p53 such as p21, Bax and Puma were significantly increased in Tenovin 6-treated UM cells (Fig. 1C).

Taken together, Tenovin-6 treatment led to accumulation of p53 protein and increased transcriptional activity of p53-regulated genes.

### Tenovin-6 suppresses the growth of UM cells

**Figure 2:**
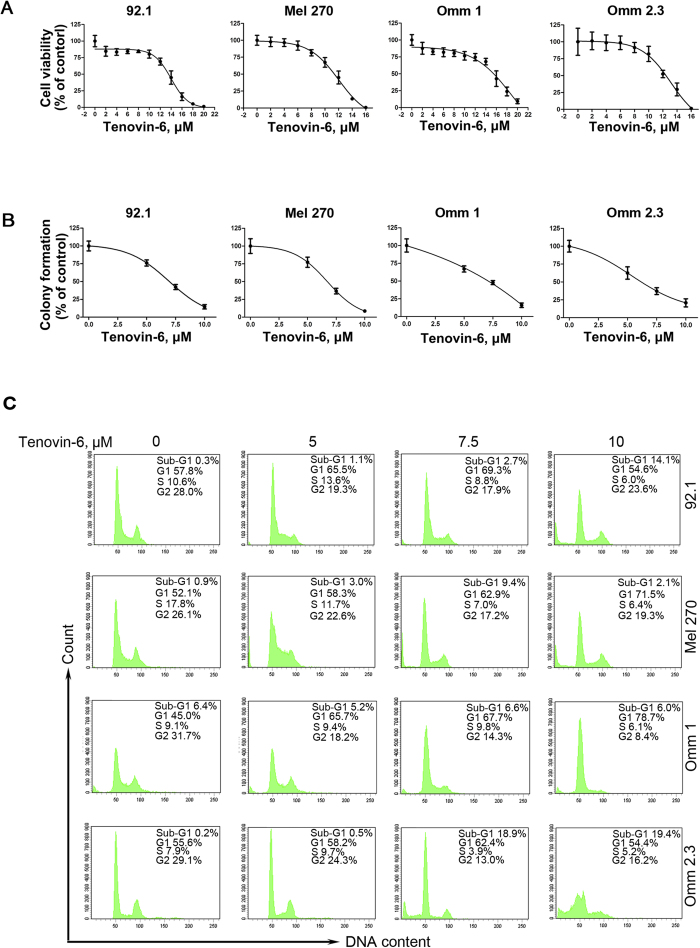
Tenovin-6 suppresses the growth of uveal melanoma cells. (**A**) UM cells were treated with escalating concentrations of Tenovin-6 for 72 h, cell viability was determined by MTS assay. (**B**) Tenovin-6 inhibited the clonogenicity of uveal melanoma cells. After treated with Tenovin-6 at the labeled concentrations for 48 h, the uveal melanoma cells were seeded in drug-free soft agar culture for 14 days, followed by counting colonies. (**C**) Effect of Tenovin-6 on cell cycle distribution in uveal melanoma cells. Cells were exposed to Tenovin-6 for 48 h, then fixed and analyzed by flow cytometry after staining with propidium iodide.

The potent effect of Tenovin-6 on p53 prompted us to evaluate its impact on cell viability of UM cells. Toward this end, 92.1, Mel 270, Omm 1 and Omm 2.3 cells were treated with increasing concentrations of Tenovin-6 for 72 h, followed by determination of the cell viability by MTS assay. As shown in Fig. 2A, the growth of UM was inhibited by Tenovin-6 in a concentration-dependent manner. The drug concentrations resulting in 50% inhibition of cell growth (IC_50_ values) of 92.1, Mel 270, Omm 1 and Omm 2.3 cells were 12.8 μM , 11.0 μM, 14.58 μM and 9.62 μM, respectively. Given that clonogenicity can better reflect malignant behavior of tumor cells, we measured the effect of Tenovin-6 on the anchorage-independent growth of 92.1, Mel 270, Omm 1and Omm 2.3 cells in soft agar culture. The results showed that Tenovin-6 inhibited the clonogenicity in a concentration-dependent fashion (Fig. 2B).

We next ascertained the effect of Tenovin-6 on cell cycle distribution in UM cells. UM cells were exposed to Tenovin-6 for 48 h, and analyzed by flow cytometry after staining with propodium iodide, the results revealed that G1 arrest was observed in Mel 270 and Omm 1 cells after Tenovin-6 treatment. However, there was not an obvious alteration in cell cycle distribution in Tenovin-6-treated 92.1 and Omm2.3 cells (Fig. 2C).

### Tenovin-6 induces apoptosis in UM cells

**Figure 3:**
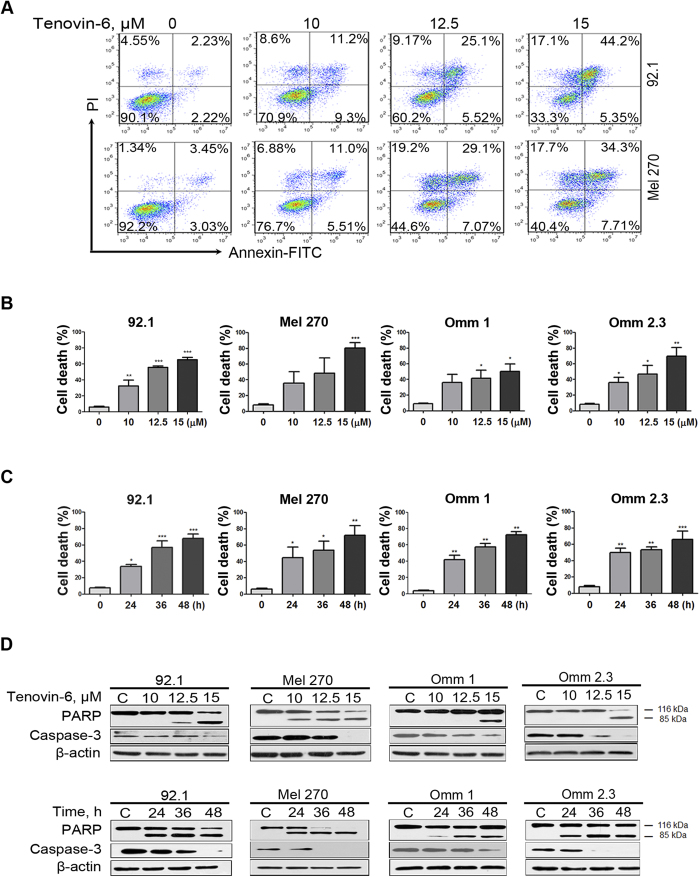
Tenovin-6 induces apoptosis in uveal melanoma cells. (**A–D**) Uveal melanoma cells were treated with increasing concentrations of Tenovin-6 for 48 h, or 15 μM Tenovin-6 for the different times, flow cytometry analysis was done after dual staining with Annexin V-FITC and PI. (**A**) Representative histograms are shown. (**B–C**) Results from 3 independent experiments are shown. The Y-axis is the sum of the top left, top right, and bottom right quadrants. (**D**) Western blotting analysis of PARP and caspase-3 in the whole-cell lysates of four UM cells are shown. The drug concentrations or incubation durations are as labeled above the gel lanes.

We next evaluated the capability of Tenovin-6 to induce apoptosis. The UM cells were treated with increasing concentrations (10 μM, 12.5 μM, 15 μM) of Tenovin-6 for 48 h or 15 μM for different durations, the cells were analyzed by flow cytometry after Annexin V-fluorescein isothiocyanate (FITC)/propidium iodide (PI) dual staining. The results indicated that Tenovin-6 treatment induced a massive apoptotic cell death in concentration- and time-dependent fashions in all four lines of UM cells (Fig. 3A–C). Western blotting analysis showed that the poly(ADP-ribose) polymerase (PARP), a hallmark of apoptosis, were specifically cleaved in concentration- and time-dependent manners in UM cells after Tenovin-6 treatment (Fig. 3D). A marked decline in precursor form of caspase-3 was observed in Tenovin-6-treated UM cells (Fig. 3D).

### Tenovin-6 decreases XIAP and survivin and leads to mitochondrial damage

**Figure 4:**
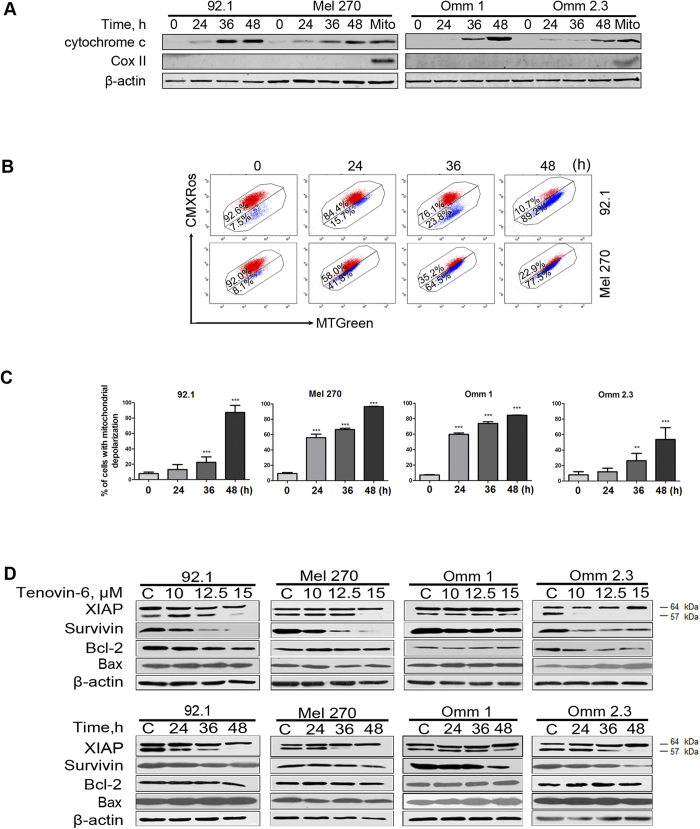
Tenovin-6 elicits cytochrome c release, mitochondrial damage. (**A**) Tenovin-6 led to release of cytochrome c from mitochondria into cytosol in UM cells. Western blotting analysis of cytochrome c in the cytosolic extracts prepared with digitonin buffer with indicated antibodies. (**B**) UM cells were treated with 15 μM Tenovin-6 for the different times as indicated then stained with CMXRos and MTGreen, and mitochondrial potential was analyzed by flow cytometry. Representative fluorescence histograms from three independent experiments are shown; (**C**) Results for 3 independent experiments are shown. (**D**) Western blotting analysis of XIAP, Survivin, Bcl-2 and Bax in the whole-cell lysates of four UM cells are shown. The drug concentrations or incubation durations are as labeled above the gel lanes.

To elucidate the mechanism of Tenovin-6-induced apoptosis, we first evaluated its impact on mitochondrial depolarization in the Tenovin-6-treated cells. Tenovin 6-treated cells also showed a remarkable increase in the levels of cytochrome c in the cytosolic extracts (Fig. 4A). The drug-treated cells were stained with CMXRos and MTGreen and underwent flow cytometry analysis of mitochondrial transmembrane potential (ΔΨm). Tenovin-6 induced a dramatic increase in the cell population with loss of ΔΨm in all four UM cell lines (Fig. 4B,C).

We also examined the apoptosis related proteins with Western blotting analysis of whole cell lysates. The results showed a decrease in XIAP and survivin but no alteration in Bcl-2 and Bax in the Tenovin 6-treated cells (Fig. 4D).

Taken together, our findings showed that tenovin-6 triggered the intrinsic apoptosis pathway.

### Tenovin-6 sensitizes UM cells to vinblastine

Because p53 can be activated by Tenovin-6 in UM cells (Fig. 1B), we evaluated the combination effect between Tenovin-6 treatment and the conventional chemotherapeutic agent vinblastine, which was used for systemic therapy of UM patients with cisplatin and dacarbazine in clinical practice^24^. 92.1 and Mel 270 cells were exposed to Tenovin-6 and vinblastine for 72 h in a serially diluted mixture (at a fixed ratio 25:1), the cell viability was then determined by MTS assay. Synergism was evaluated by the median effect method of Chou and Talalay^25^. As shown in Figure S1A, the combination index <1 indicated that the combination of Tenovin-6 and vinblastine synergistically inhibited the viability of UM cells, and Western blotting analysis of specific cleavage of PARP further showed enhanced ability of two drug combination to induce apoptosis in UM cells (Figure S1B).

### Tenovin-6 increases introcellular ROS level and contributes to apoptosis in UM cells

**Figure 5:**
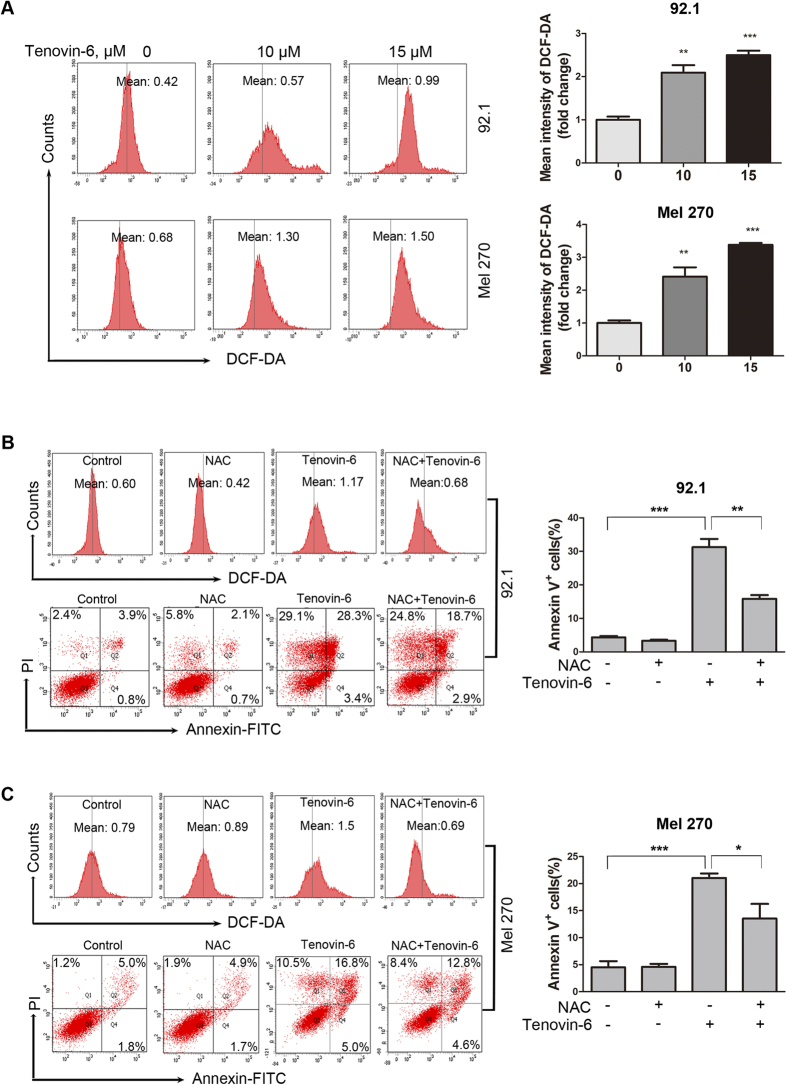
Tenovin-6 increases ROS level in UM cells. (**A**) UM cell line 92.1 and Mel 270 were treated with 10 μM and 15 μM for 24 hours, incubated with 2.5 μM CM-H2DCF-DA for 60 min. The results were analyzed by flow cytometry for 3 independent experiments. (**B–C**) *Left, top.* 92.1 and Mel 270 cells were incubated with Tenovin-6(15 μM) ± NAC for 24 hours, and then subjected to flow cytometry. *Left, bottom.* The cells were detected by flow cytometry after stained with Annexin V-FITC and PI. *Right.* The percentage of Annexin V^+^ cells were results for 3 independent experiments. + indicates the presence and −indicates the absence of the respective drugs.

HDACis were reported to increase reactive oxygen species (ROS) to kill tumor cells^26^,^27^, we therefore assessed whether Tenovin-6 affected intracellular ROS levels. The results revealed that the intracellular ROS levels were increased in the UM cells exposed to 10 μM and 15 μM of Tevonin-6 for 24 hours (Fig. 5A).

We next determined the role of Tenovin-6-induced ROS elevation in its cytotoxicity. Pre-treatment with 20 mM N-Acetylcysteine (NAC) completely abrogated the Tenovin-6-mediated ROS elevation (Fig. 5B,C, *left, top*), however, only led to attenuation of apoptotic cell death in Tenovin-6-treated 92.1 and Mel 270 cells (Fig. 5B–C, *left, bottom and right*). Collectively, ROS elevation may partially contribute to the Tenovin-6-induced apoptosis.

### Tenovin-6 attenuates migration of UM cells

**Figure 6:**
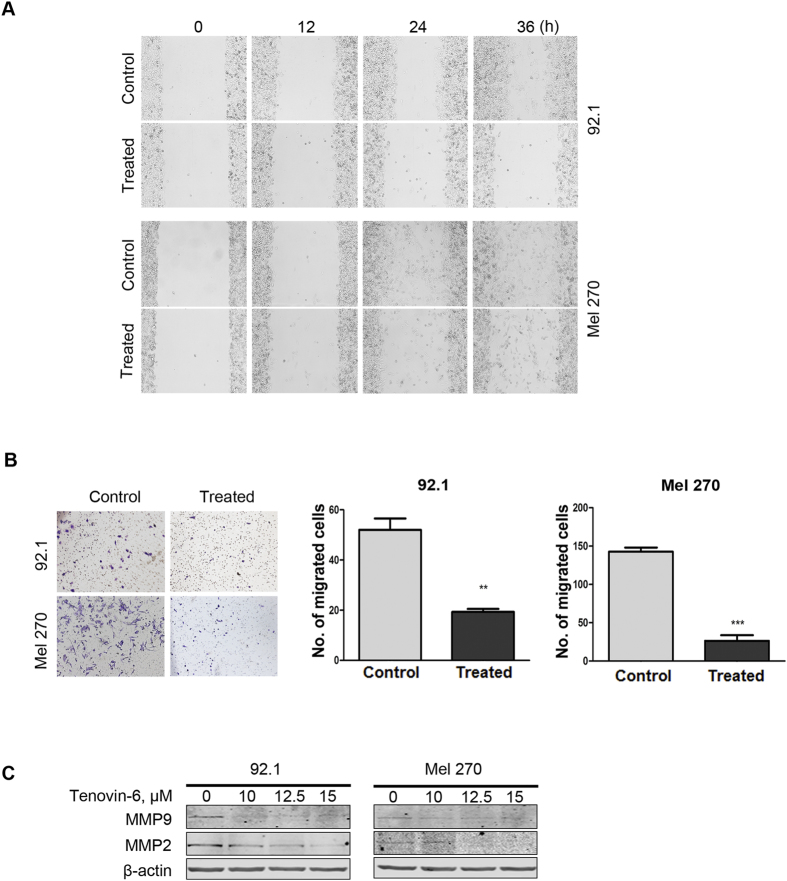
Tenovin-6 blocks the migration in UM cells . (**A**) Wound-healing scratch assay in Mel 270 and 92.1 after exposed to control medium or Tenovin-6 (10 μM). (**B**) Mel 270 and 92.1 cells were exposed to Tenovin-6 (10 μM) for 48 h, then seeded in transwells for 2 days. Migrated cells were counted after fixation with formaldehyde and staining with crystal violet. (**C**) Results from 3 random fields are shown. (**D**) Mel 270 and 92.1 were exposed to increasing concentrations of Tenovin-6 for 48 h, Western blotting analysis was conducted with indicated antibodies.

Despite effective therapeutic approaches for treating primary tumors in the eye, the mortality rate for UM has not changed, perhaps due to severely limited therapeutic options to prevent metastases^28^. To investigate the effect of Tenovin-6 on migration of UM cells, 92.1 and Mel 270 cells were subjected to wound-healing scratch assay. The cells were cultured in a medium with 10% FBS for 24 h, confluent monolayer of these cells was scratched and then cultured in a medium with different concentrations of Tenovin-6 for ∼36 h. After scratching at time 0 h, the wounded area was monitored by inverted phase-contrast microscopy microscopy at 12, 24, 36 h post-scratch. The scratch healing in 92.1 and Mel 270 cells were significantly inhibited by Tenovin-6 (Fig. 6A). Further quantitative analysis with transwell (Boyden chamber) assay showed that Tenovin-6 reduced the migration ability of 92.1 and Mel 270 cells (Fig. 6B). Moreover, Western blotting analysis showed that matrix metalloproteinase (MMP) 2 and MMP9, which play a critical role in invasion and metastasis^29^,^30^, were decreased in Tenovin-6-treated 92.1 and Mel 270 cells (Fig. 6C). Taken together, these data suggested that Tenovin-6 could inhibit cell migration.

### Tenovin-6 decreases cancer stem cells in UM cells

**Figure 7:**
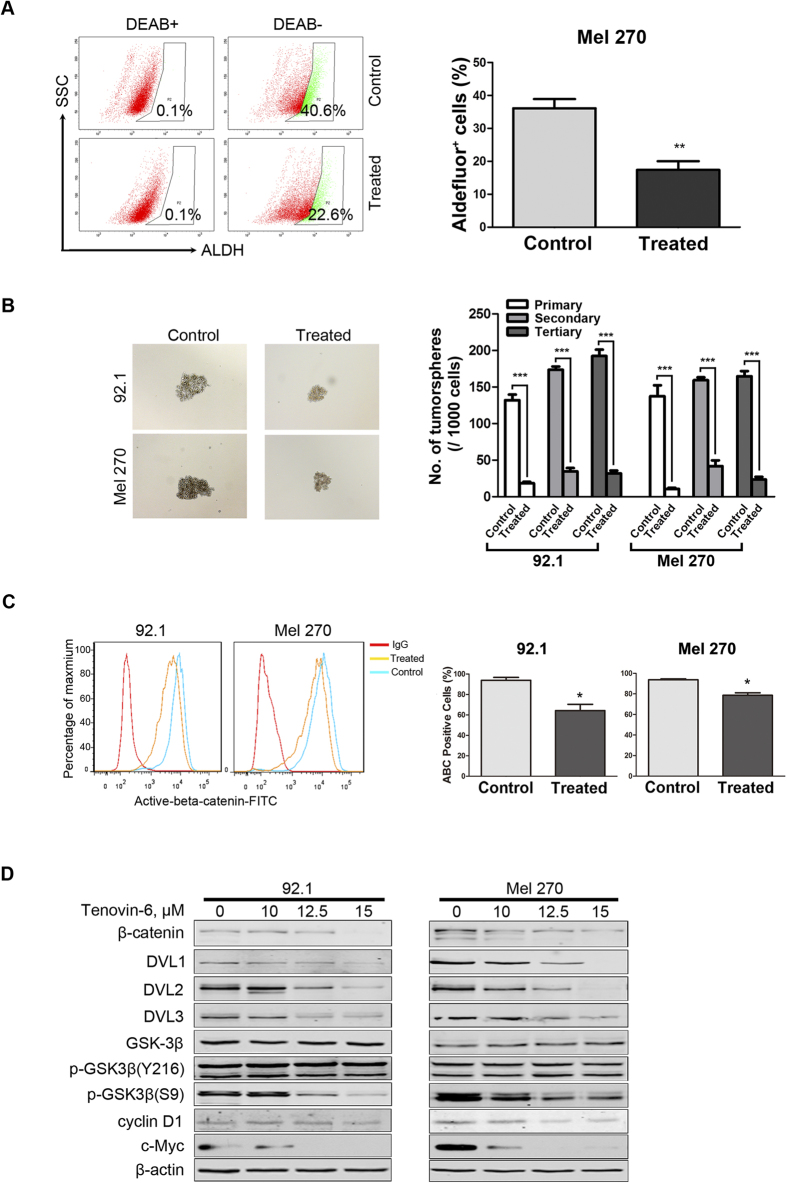
Tenovin-6 inhibits properties of cancer stem cells through lowering β-catenin in UM cells. (**A**) Mel 270 cells were was exposed to 0 μM (control) or 10 μM (treated) Tenovin-6 for 48 h, the ALDH activity was detected by flow cytometry. *left,* representative flow cytometry results from 3 independent experiments are shown. *right*, quantitive analysis (*Right*) of ALDH^+^ cells are shown. (**B**) Tenovin-6 inhibited the formation of tumor spheres. Mel 270 and 92.1 cells were treated with 0 μM (control) or 10 μM (treated) Tenovin-6 for 48 hours and then cultured with the DMEM/F-12 medium for 14 days, tumor sphere units were counted. (**C**) *Left.* Tenovin-6 reduced the active-β-catenin in UM cells. 92.1 cells and Mel 270 cells were incubated with 10 μM Tenovin-6 for 24 hours and then stained with the antibody of active-beta-catenin and analyzed by flow cytometry. *Right.* Results from 3 random fields are shown. (**D**) The effect of Tenovin-6 on Wnt/β-catenin signaling pathway. Mel 270 and 92.1 cells were treated with increasing concentrations of Tenovin-6 for 48 hours. Cell lysates were harvested and detected by Western blotting with the indicating antibodies.

In recent years, a growing body of evidence supports the hypothesis that tumor initiation as well as therapy resistance may be caused by the presence of cancer stem cells (CSCs)^31^,^32^. Aldehyde dehydrogenase (ALDH) has been a functional marker for identification of cells with stem cell properties in several cancers^33^,^34^. We assessed the effect of Tenovin-6 on ALDH^+^ cells with flow cytometry. The results showed that percentage of ALDH^+^ cells in Mel 270 were significantly decreased after 10 μM Tenovin-6 treatment (Fig. 7A, p<0.01, Student’s t test). We next investigated the effects of Tenovin-6 on tumor sphere formation. After exposed to 10 μM Tenovin-6 for 48 h, 92.1 and Mel 270 cells were harvested, washed and seeded into 24-well ultra-low attachment plates with drug-free DMEM/F-12 medium for 10–14 days. The sizes and numbers of the tumor spheres were decreased in Tenovin 6-treated cells (Fig. 7B). Three rounds of replating experiments revealed that the serial replating ability was also significantly decreased in Tenovin 6-treated cells (Fig. 7B). These data suggested that Tenovin-6 may be active against CSCs in UM cells.

### Tenovin-6 lowers the intracellular active-β-catenin and blocks Wnt/β-catenin signaling in UM cells

Wnt/β-catenin cascade is one of the important intrinsic pathways to regulate self-renewal of cancer stem cells^35^. Previous studies showed that p53 can down-regulate β-catenin protein level^36^, and SIRT1 regulates Wnt/β-catenin pathway by binding to β-catenin and Dishevelled (Dvl) proteins^37^. We therefore investigated whether Tenovin-6 inhibited the Wnt/β-catenin pathway in UM cells. 92.1 and Mel 270 cells were exposed to 10 μM Tenovin-6 for 24 h and stained with active-β-catenin antibody and corresponding FITC-conjugated secondary antibody, then subjected to flow cytometry analysis of the percentage of active-β-catenin-positive cells. The results revealed that Tenovin-6 reduced active-β-catenin-positive cells compared with the control (Fig. 7C). We next determined the effect of Tenovin-6 on Wnt/β-catenin signaling in UM cells. 92.1 and Mel 270 cells were treated with different concentrations of Tenovin-6 for 48 hours and then showed a concentration-dependent decrease in β-catenin protein when the whole cell lysates were analyzed by Western blotting (Fig. 7D). Consistently, the expression of c-Myc and cyclin D1, two key downstream targets of β-catenin were also lowered in the Tenovin-6-treated cells (Fig. 7D).

Western blotting analysis of upstream components of Wnt/β-catenin cascade showed that the phosphorylation at S9 in GSK3β (inactive GSK3β) was attenuated with no change of phosphorylation at Y216 (active GSK3β) and total GSK3β protein in Tenovin 6-treated cells (Fig. 7D). Moreover, the protein levels of DVL1, DVL2, DVL3 protein (Fig. 7D) were strikingly lower in 92.1and Mel 270 cells treated with Tenovin-6 compared with those in the untreated control cells. Together, these results suggest that Tenovin-6 inhibits Wnt/β-catenin signaling perhaps through repressing GSK3β (S9) and DVL protein.

## Discussion

Little is known about effective therapeutic targets in UM. In the present study, we discovered that targeting class III HDACs SIRT1/2 by Tenovin-6 induced massive apoptosis in UM cells by enhancing the expression of tumor suppressor genes p53 and elevating intracellular ROS. Our data revealed that Tenovin-6 might be a promising agent for the treatment of UM.

We documented here that Tenovin-6 effectively inhibited cellular proliferation and induced apoptosis in four UM cell lines as a single agent through inhibiting the deacetylation activity of SIRT1/2. These results were consistent with the effects of HDACis on other types of cancer cells^38^,^39^. Vinblastine is one of the standard chemotherapy agents for UM, and the combination of Tenovin-6 and vinblastine was synergistic. Furthermore, we found that Tenovin-6 notably reduced the migration ability of UM cells (92.1 and Mel 270) along with inhibition of expression of MMP9 and MMP2. The antitumor activity of Tenovin-6 in UM is consistent with the previous observations in acute myeloid leukemia^40^, acute lymphoblastic leukemia^21^, breast cancer^41^, prostate tumors^42^,^43^ and colorectal cancer^44^.

In contrast to other tumor types, mutation of tumor suppress gene p53 is not common in UM. p53 gene rarely has mutation in patients with UM and UM cell lines including 92.1, Mel 270, Omm 1 and Omm 2.3 45,46,47. Given that the elevated wild type p53 has potent activity to induce apoptosis and cell cycle arrest, and suppress metastasis of tumor cells, our observed effect of Tenovin-6 on UM cells can be explained at least in part by induction of p53. The decrease in XIAP and survivin (Fig. 4D) and the ROS generation (Fig. 5) may contribute to Tenovin-6-induced apoptosis.

CSCs are considered to be responsible for the resistance to conventional therapies, metastatic abilities, and the source of relapse of tumor. Although the biomarkers of CSCs in UM have not been well established, CSCs can be analyzed by evaluating the ALDH^+^ cells and tumor sphere formation. In the present study, we showed that Tenovin-6 treatment significantly decreased the population of ALDH^+^ cells and tumor sphere formation ability. Therefore, we conclude that Tenovin-6 eliminates CSCs in UM. The anti-CSCs effect is consistent with that in leukemias^20^,^21^.

Multiple alterations caused by Tenovin-6 treatment may contribute to elimination of CSCs in UM. First, Wnt/β-catenin pathway is a key intrinsic regulator of self-renewal of CSCs^35^. Our results revealed that Tenovin-6 suppressed the expression of β-catenin. Dvl proteins are located at upstream of β-catenin positively regulating at least two layers: recruiting destructive box components to degrade β-catenin and increasing TEF/LEF transcriptional activity by physical binding with β-catenin. It is plausible that the lowered Dvl protein by Tenovin-6 treatment might facilitate elimination of UM CSCs. In addition, the elevated p53 and decreased c-Myc rendered by HDAC inhibitors may constitute negative forces against self-renewal of CSCs^48^.

In summary, the novel SIRT1/2 inhibitor Tenovin-6 is effective to induce apoptosis in UM cells and eliminate CSCs. Tenovin-6 may represent an important potential therapeutic agent alone or in combination with standard chemotherapy against UM, and is therefore worthy of further clinical investigation for UM treatment.

## Materials and Methods

### Chemicals and antibodies

Tenovin-6 (chemical structure, Fig. 1A) was purchased from Cayman Chemical (Ann Arbor, MI). Annexin V–FITC and N-acetylcysteine (NAC) were from Sigma-Aldrich (Shanghai, China). CM-H2DCF-DA and antibody against cytochrome c oxidase subunit II (COXII) were purchased from Invitrogen (Shanghai, China). Antibodies against SIRT1 (H-300), p53 (DO-7), Bax, DVL1, DVL3, β-catenin, cyclin D1 (C-20), survivin and c-Myc were from Santa Cruz Biotech. (Santa Cruz, CA). Antibody against active-β-catenin was from Millipore and the fluorescent secondary antibody was purchased from life technologies (Eugene. OR). Antibodies against PARP (clone 4C10-5), caspase-3, cytochrome c (clone 6H2.B4), XIAP, Bcl-2, phospho-GSK3β (Y216) were purchased from BD Biosciences (San Jose, CA). Antibodies against K382-acetyl-p53, DVL2, phospho-GSK3β (S9), MMP2 and MMP9 were from Cell Signaling Tech. (Beverly, MA). Anti-SIRT2 was purchased from Atlas Antibodies. Anti-mouse immunoglobulin G and anti-rabbit immunoglobulin G fluorescent-conjugated secondary antibodies were from LI-COR Biotechnology (Nebraska, USA).

### Cell culture

The UM cell lines 92.1, Mel 270, Omm 1 and Omm 2.3 cells were generously provided by Dr. M. J Jager, Leiden University Medical Center, Leiden, The Netherlands. The cells were cultured in RPMI 1640 supplemented with 10% FBS in a 37 °C humidified incubator containing 5% CO_2_^49^.

### Cell viability assay

The MTS assay (CellTiter 96Aqueous One Solution reagent; Promega) was used to evaluate cell viability. UM cells were seeded into each well of 96-well plates (5,000 cells/well) and treated the next day with control or Tenovin-6 in an increasing concentrations from 0 to 20 μM for 68 h, and then MTS was added at 20 μl/well^50^,^51^ to be read at a wave length of 490 nm, the IC_50_ was determined by curve fitting of the sigmoidal dose-response curve.

### Colony-formation assays

UM cells were treated with increasing concentrations of Tenovin-6 at 0 ∼ 10 μM or diluent (DMSO, control) for 48 h, harvested and washed, and then 5000 cells were incubated in a modified drug-free double layer soft agar system^52^. After incubation for 10 to 14 days at 37 °C, colonies composed of >50 cells were counted using an inverted phase-contrast microscope.

### Real-time quantitative RT-PCR

Total mRNA was extracted with Trizol reagent (Invitrogen), then reverse-transcribed into first-strand complementary DNA (cDNA) with maxima first strand cDNA synthesis kit (Thermo Fisher). Expression levels of GAPDH were used as an endogenous reference.

PCR primers were as follows: *p21,* forward 5′-GACTCTCAGGGTCGAAAACGG-3′, reverse 5′-GCGGATTAGGGCTTCCTCTT-3′; *Bax*, forward 5′-GAACCATCATGGGCTGGACA′, reverse 5′-GCGTCCCAAAGTAGGAGAGG′; *Puma,* forward 5′-ACCTCAACGCACAGTACGAG-3′, reverse 5′-CGGGTGCAGGCACCTAATTG′; GAPDH, forward 5′-GATCGAATTAAACCTTATCGTCGT-3′, reverse 5′-AGCAGCAGAACTTCCACTCGGT-3′

The reaction was performed in SYBR Premix Ex Taq (Perfect Real-time; Takara Bio) with BIO-RAD CFX96 Real-Time Thermocycler (CFX96, Bio-Rad Laboratories, Hercules, CA) according to the manufacturer’s instructions.

### Western blotting analysis

Whole cell lysates were prepared in RIPA buffer (1×PBS, 1% NP-40, 0.5% sodium deoxycholate, 0.1% SDS). For detection of cytochrome c, cytosolic fraction was prepared in digitonin extraction buffer (10 mM PIPES, 0.015% digitonin, 300 mM sucrose, 100 mM NaCl, 3 mM MgCl_2_, 5 mM EDTA, and 1 mM phenylmethylsulfonyl fluroride). 1×protease inhibitor cocktail (Roche), 10 mM β-glycerophosphate, 1 mM sodium orthovanadate, 10 mM sodium fluoride, and 1 mM phenylmetnylsulfonyl fluoride were added to the buffers mentioned above. Equal amounts of protein samples were separated by SDS–PAGE gel electrophoresis and then transferred to nitrocellulose membranes, which were then incubated with the primary antibodies overnight. The β-actin was used as the internal control of protein loading. After incubation with appropriate secondary antibodies, the immunoblots were recorded with the Odyssey infrared imaging system (LI-COR).

### Flow cytometry analysis of cell cycle

UM cells were treated with different concentrations of Tenovin-6 for 48 h, and then collected and fixed overnight with 66% cold ethanol at 4 °C. The cells were then washed twice in cold PBS and labeled with propidium iodide for 1 hour in the dark. Cell cycle distribution was determined by a FACS LSRFortessa flow cytometer with its software^51^.

### Apoptosis assay by flow cytometry

Apoptosis was measured by Annexin V-fluoresceinisothiocyanate (FITC) and propidium iodide apoptosis detection kit (Sigma-Aldrich, Shanghai) according to the instructions of the manufacturer and analyzed with a FACS C6 flow cytometer^53^.

### Measurement of mitochondrial transmembrane potential

UM cells were exposed to 15 μM Tenovin-6 for different durations, then the cells were subjected to flow cytometry to examine the changes in inner mitochondrial transmembrane potential (ΔΨm) after incubation with submicromolar concentrations of MitoTracker probes (CMXRos and MTGreen, Eugene, OR, USA) as described previously^54^.

### Assessment of intracellular reactive oxygen species

The intracellular reactive oxygen species (ROS) contents were detected after staining with CM-H2DCF-DA by flow cytometry analysis using a FACS LSRFortessa as described previously^55^.

### Wound-healing scratch assay

After cultured in a RPMI160 medium with 10% FBS for 24 h, the confluent UM cell monolayer of each plate was scratched at the same size and then cultured in a medium with 0 μM (control) or 10 μM (treated) Tenovin-6 for 36 h. The same wounded area was measured by inverted phase-contrast microscope at 0, 12, 24, 36 h post-scratching^56^.

### Aldehyde dehydrogenase (ALDH) assay

The Aldefluor kit (Stem Cell Technologies, Vancouver, BC, Canada) was used to identify the effect of Tenovin-6 on cell populations with ALDH enzymatic activity. Briefly, Mel270 cells were treated with 0 μM (control) or 10 μM (treated) Tenovin-6 for 48 h, 10^6^ cells were harvested from cell cultures, and then operated according to the instructions of the manufacturer and analyzed with use of a FACS LRSFortessa flow cytometer^57^.

### Tumor sphere culture

92.1 and Mel270 cells were treated with 0 μM (control) or 10 μM (treated) Tenovin-6 for 48 h, harvested, washed, then 5000 cells were seeded in the DMEM/F-12 medium (HyClone, containing B27 1 ml, bFGF 10 ng/ml, EGF 20 ng/ml) to each well of 24-well Corning^TM^ Ultra-Low Attachment Plates (Thermo Fisher Scientific Inc., Waltham, MA) for 10–14 days without disturbing the plates and without replenishing the medium. The secondary and tertiary rounds of tumor sphere culture were similary conducted after harvesting the first round of tumor sphere culture cells. At the end of each round of culture, the number and size of tumor spheres were determined^58^.

### Measurement of active-β-catenin by flow cytometry

After cultured in complete medium containing 10 μM Tenovin-6 for 24 h, the cells were harvested, and fixed, and then incubated with the primary antibody of active-β-catenin and corresponding FITC-conjugated secondary antibody, respectively. The cells were analyzed by FACS LRSFortessa flow cytometer at 488 nm.

### Statistics

Data from all the experiments were expressed as mean ± standard error of the mean (SEM). GraphPad Prism 5.0 software (GraphPad Software, San Diego, CA) was used for statistical analysis. Difference between two groups was tested by 2-sided Student’s *t* test; the analyses of experiments with multiple groups were performed by one-way analysis of variance (ANOVA) with *post-hoc* intergroup comparisons with the Tukey test. P < 0.05 was considered to be a statistically significant difference. The experimental data were from at least three independent experiments.

## Additional Information

**How to cite this article**: Dai, W. *et al.* Class III-specific HDAC inhibitor Tenovin-6 induces apoptosis, suppresses migration and eliminates cancer stem cells in uveal melanoma. *Sci. Rep.* **6**, 22622; doi: 10.1038/srep22622 (2016).

## Supplementary Material

Supplementary Information
