# Supplementary material for: Class III-specific HDAC inhibitor Tenovin-6 induces apoptosis, suppresses migration and eliminates cancer stem cells in uveal melanoma
Source: Sci Rep. 2016 Mar 4;6:22622. doi: 10.1038/srep22622 (PMC4778058; doi:10.1038/srep22622)
Supplement: Supplementary Information [file srep22622-s1.pdf]

Class III-specific HDAC inhibitor Tenovin-6 induces apoptosis, suppresses migration and eliminates cancer stem cells in uveal melanoma

Wei Dai,[1] Jingfeng Zhou,[1] Bei Jin,[1] Jingxuan Pan[1,2]

[1]State Key Laboratory of Ophthalmology, Zhongshan Ophthalmic Center, Sun Yat-sen University; Jinan University Institute of Tumor Pharmacology, Guangzhou, China

[2]Collaborative Innovation Center for Cancer Medicine, State Key Laboratory of Oncology in South China, Sun Yat-Sen University Cancer Center, Guangzhou, China

Corresponding authors:

Jingxuan Pan, MD, Ph.D, State Key Laboratory of Ophthalmology, Zhongshan Ophthalmic Center, Sun Yat-sen University, 54 South Xianlie Road, Guangzhou 510060, People's Republic of China, Phone: +86-20-37628262, Email: panjx2@mail.sysu.edu.cn

Supplementary Figure S1

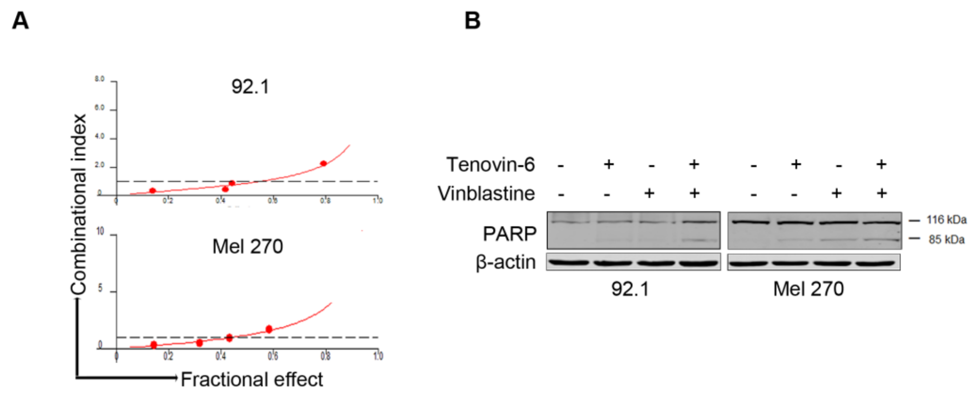

Supplementary Figure S1. Tenovin-6 displays synergism with vinblastine in UM cells. **(A)**Synergistic effect of the combination of Tenovin-6 and vinblastine in Mel 270 and 92.1 was assessed by MTS assay after incubation with a serial diluted mixture (at a fixed ratio of Tenovin-6:Vinblastine = 25:1) for 72 h. The combination index (CI) was the ratio of the combination dose to the sum of the single-agent doses at an isoeffective level. A reference line is drawn at CI = 1. CI values of <1 indicated synergism between the 2 drugs. **(B)**Mel 270 and 92.1 cells of four UM cells are shown. The drug concentrations or incubation durations are as labeled above the gel lanes.were exposed to Tenovin-6 (5 μM) with combination of vinblastine (150 nM) for 48 h, the cleavage of PARP was detected by Western blotting. + indicates the presence and – indicates the absence of the respective drugs.
